# Graves' Disease Following COVID-19 Vaccination

**DOI:** 10.7759/cureus.24418

**Published:** 2022-04-23

**Authors:** Gurdeep Singh, Timothy Howland

**Affiliations:** 1 Endocrinology, Diabetes and Metabolism, Our Lady of Lourdes Memorial Hospital, Binghamton, USA; 2 Internal Medicine/Endocrinology, Our Lady of Lourdes Memorial Hospital, Binghamton, USA

**Keywords:** mrna vaccine, covid-19 vaccine, graves´disease, johnson and johnson vaccine, moderna vaccine, covid 19

## Abstract

Autoimmune endocrine diseases have been reported after influenza and the human papillomavirus vaccine, but there is limited data on autoimmune diseases after coronavirus disease 2019 (COVID-19) vaccination. Our report is about a 42-year-old Caucasian male and a 68-year-old Caucasian female who developed Graves’ disease after receiving Moderna (Moderna, Inc., Cambridge, Massachusetts, United States) and Johnson & Johnson (Johnson & Johnson, New Brunswick, New Jersey, United States) vaccines, respectively. Both patients had no previous autoimmune thyroiditis and had normal thyroid function but developed hyperthyroidism characterized by suppressed thyroid-stimulating hormone (TSH), elevated free T4 level, and TSH receptor antibodies after vaccination.

COVID-19 vaccines, either mRNA-based (Moderna) or non-mRNA-based (Johnson & Johnson), can cause Graves’ disease. The clinical manifestations are similar to Graves’ disease but without ocular manifestations.

## Introduction

The coronavirus disease 2019 (COVID-19) pandemic started in late 2019, with the first case being reported in Wuhan, China [ 1]. The angiotensin-converting enzyme 2 (ACE2) receptor has been considered the intracellular entry point of the COVID-19 virus. Widespread expression of the ACE2 receptor may explain the observation that COVID-19 infection frequently involves multiple organs [2]. 

In addition to direct organ involvement by the virus, COVID-19 vaccines have been implicated in the precipitation of autoimmune thyroiditis with or without the presence of anti-thyroid antibodies [3-10], but only a few cases of Graves’ disease have been reported following vaccination [11-13]. The autoimmune/inflammatory syndrome induced by vaccine adjuvants (ASIA) involves a spectrum of autoimmune diseases [11]. The widely used adjuvants are oils, lipopolysaccharides, mineral salts, and peptidoglycans [11]. These have been associated with autoimmune endocrinopathies, including Addison’s disease, Graves’ disease, Hashimoto’s thyroiditis, type 1 diabetes, and ovarian failure [11].

In the United States, the Food and Drug Administration approved two mRNA bases vaccines, Pfizer (Pfizer Inc., New York, United States) and Moderna (Moderna, Inc., Cambridge, Massachusetts, United States), and one adenoviral vector vaccine (non-MRNA-based), Johnson & Johnson (Johnson & Johnson, New Brunswick, New Jersey, United States). We are reporting two cases of Graves’ disease after the COVID-19 vaccination. We did not find any literature regarding previous reports of Graves’ Disease following the Moderna vaccine or the Johnson & Johnson vaccine.

## Case presentation

Case 1

A 42-year-old Caucasian male with no previous history of thyroid disease received a Moderna booster on November 3, 2021; two days later, he developed nausea, significant muscle weakness, shortness of breath on exertion, excessive sweating, headache, and difficulty sleeping at night. He lost 20 pounds within 11 days of his vaccination.

**Figure 1:**
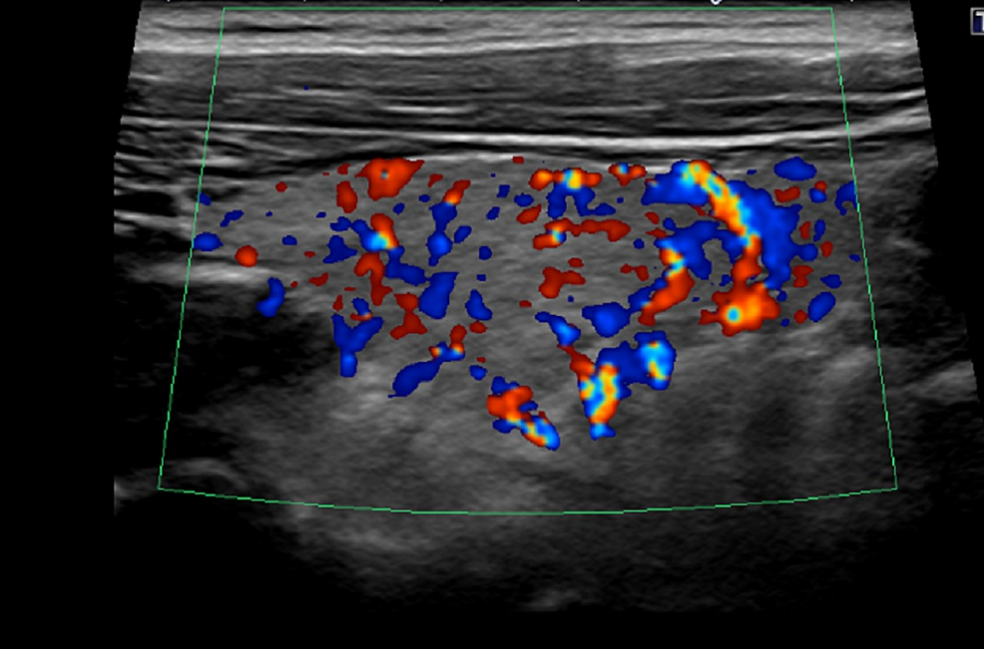
Thyroid ultrasound of Case 1: long axis with doppler showing increased vascularity in the entire right lobe

**Figure 2:**
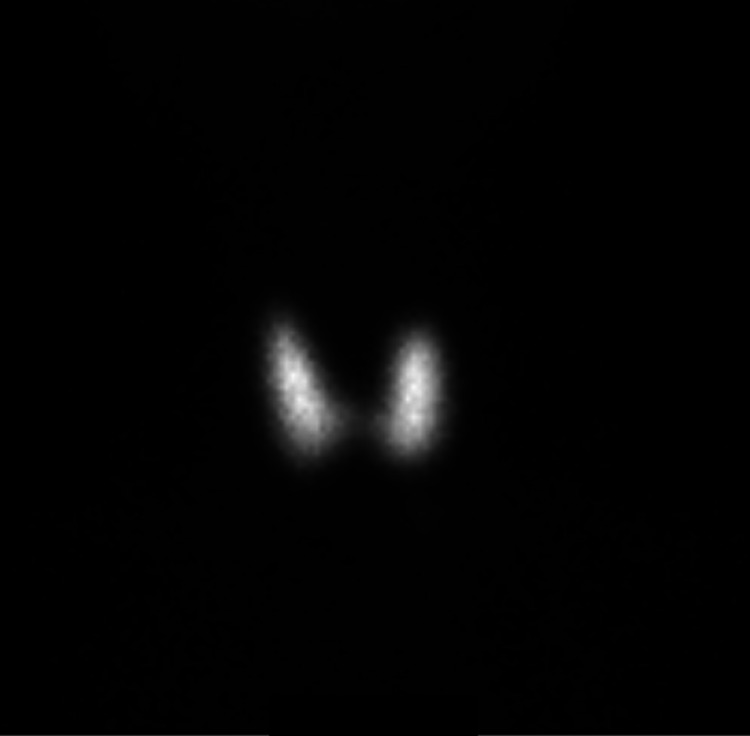
Thyroid uptake and scan with technetium 99 pertechnetate showing avid uptake in Case 1

Physical examination revealed tachycardia with a heart rate of 110/min at rest. Laboratory tests (Table 1) showed that WBC count was 4.1 k/microliter (4-10.5), Influenzavirus A and B were negative, Covid-19 polymerase chain reaction (PCR) was negative, C-reactive protein was less than 0.9. Thyroid studies showed TSH less than 0.015 mcIU/ml (reference range 0.45-4.5 mcIU/ml) and free T4 5.96 (reference range 0.78-2.19 ng/dl). TSH receptor antibody was markedly elevated at 16.1 IU/L (reference range <1.75 IU/L) and microsomal antibodies were elevated at 70.25 (reference range <5.6 IU/ml). Thyroid ultrasound showed a prominent heterogeneous and hyperemic gland (Figure 1). Thyroid scan showed bilateral avid symmetric radionuclide uptake consistent with a hyperfunctioning gland (Figure 2). The patient was started on methimazole and propranolol.

**Table 1:** Laboratory results of Case 1 TSR: thyroid-stimulating hormone; CRP: C-reactive protein; COVID-19: coronavirus disease 2019; PCR: polymerase chain reaction

	At diagnosis	Reference range
TSH	< 0.015	0.45-4.5 mcIU/ml
Free T4	5.96	0.78-2.19 ng/dl
TSH Receptor Ab	16.1	<1.75 IU/L
Thyroid peroxidase Ab	70.25	<5.6 IU/ml
CRP	< 0.9	
COVID-19 PCR	Negative	

Case 2

A 68-year-old female without previous history of thyroid disease and a normal thyroid function with a TSH level of 0.77 mIU/ml (0.45-4.5) on February 25, 2019, received the Johnson & Johnson vaccine on March 6, 2021. She developed a constellation of nonspecific symptoms within 24-48 hours after receiving the vaccine. Blood work on March 9, 2021, showed TSH 0.23 mIU/ml (0.45-4.5), free T4 1.41 ng/dl (0.82-1.77), WBC 4.4 K/microliter (3.4-10.8) (Table 2).

**Table 2:** Laboratory results of Case 2 NA: not available; TSH: thyroid-stimulating hormone;

	At diagnosis (Reference Range)	After one month (Reference Range)
TSH	0.23 (0.45-4.5 mIU/ml)	< 0.01 (0.45-4.5 mIU/ml)
Free T4	1.41 (0.82-1.77 ng/dl)	3.6 (0.6-1.3 ng/dl)
Free T3	NA	13.8 (2.5-3.9 pg/dl)
TSH Receptor Ab	NA	14.3 (< 1.75 IU/L)
Thyroid peroxidase Ab	NA	5.84 (< 5.61 IU/ml)

**Figure 3:**
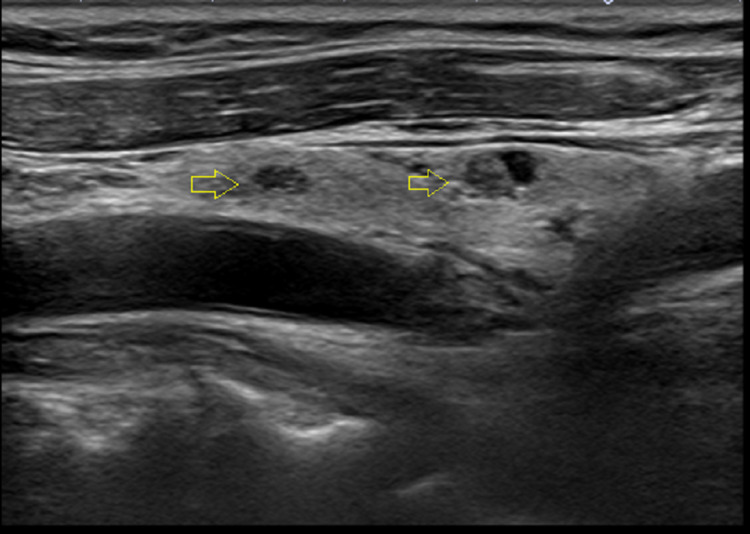
Thyroid ultrasound in Case 2: long axis showing small benign-appearing nodules in the right lobe

**Figure 4:**
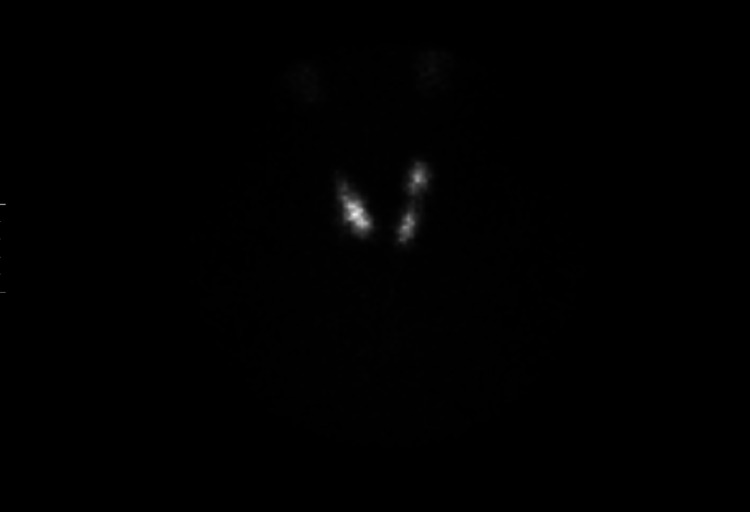
Thyroid uptake and scan with technetium 99 pertechnetate showing normal uptake in Case 2

She was re-evaluated on April 7, 2021, and was found to be in atrial fibrillation. Her history was most remarkable for a relative paucity of hyperthyroid symptoms and no eye signs of Graves’ disease. She had a suppressed TSH, free T4 3.6 ng/dl ( 0.6-1.3), and free T3 13.8 pg/ml (reference range 2.5-3.9). Thyroid ultrasound showed subcentimeter thyroid nodules in the right and left thyroid lobe that corresponded to the area with decreased technetium uptake on the scan (Figure 3). A technetium thyroid scan showed inappropriately normal uptake bilaterally with a region of relative photon deficiency in the right and left thyroid lobes due to nodules (Figure 4). She was started on methimazole, beta-blocker, and apixaban due to the new onset of atrial fibrillation.

## Discussion

The appearance of autoimmune diseases after vaccination has been described [14]. Autoimmune endocrine disorders have been reported after influenza and human papillomavirus vaccines [15], but there is limited data on autoimmune diseases after COVID-19 vaccines. 

Vera-Lastra et al. reported two cases of Grave disease, three days after vaccination with Pfizer-BioNTech (Pfizer Inc., New York, United States, and BioNTech SE, Mainz, Germany), both were found to have positive thyroid receptor antibodies and thyroid microsomal antibodies along with hyperthyroidism [11]. Zettinig et al. reported two cases of Graves’ disease after the Pfizer-BioNTech vaccine [12]. The first patient was a 71-year-old female who had a previous Graves' disease history, in remission with negative thyroid receptor antibodies. She developed thyrotoxicosis and positive thyroid receptor antibodies, one month after the second dose. The second case was a 46-year-old male with no prior history of Graves’ disease. He developed thyrotoxicosis and positive thyroid receptor antibodies two weeks after the first dose. Chutinform Sriphrapradang reported a case of a 30-year-old female with worsening Graves’ disease after a booster with a replication-deficient chimpanzee adenovirus-vectored vaccine ChAdOx1 nCoV19 (AZD 1222; Oxford-AstraZeneca, University of Oxford, Oxford, United Kingdom, and AstraZeneca plc, Cambridge, United Kingdom) [13].

The onset of autoimmune diseases following COVID-19 vaccination could occur due to the adjuvants in the vaccine. ASIA involves a spectrum of autoimmune diseases. [14]. It was initially described in 2011 by Shoenfield et al. [16]. Adjuvants are commonly used in vaccines to potentiate the immunogenic response against the inoculated antigen but can lead to autoimmune diseases in genetically susceptible people [17]. The widely used adjuvants are oils, lipopolysaccharides, mineral salts, and peptidoglycans. These have been associated with autoimmune endocrinopathies, including Addison’s disease, Graves’ disease, Hashimoto’s thyroiditis, type 1 diabetes, and primary ovarian failure [18].

Another possible mechanism of autoimmune diseases after COVID-19 vaccination is the molecular mimicry between the severe acute respiratory syndrome coronavirus 2 (SARS-CoV-2) spike glycoprotein and human proteosomes and thyroid peroxidase [19]. In addition, cross-reactivity can damage various organs, including the thyroid gland, due to peptide sharing [19].

The exact mechanism of autoimmune endocrine diseases after Moderna and Johnson & Johnson vaccination is unknown. Polyethylene glycol, which may cause an autoimmune response, is used as an adjuvant in the Moderna and Pfizer vaccine but not in Johnson & Johnson vaccine.

## Conclusions

To our knowledge, this is the first case report of Graves’ disease after Moderna and Johnson & Johnson vaccines. The overall incidence of Graves’ disease after the COVID-19 vaccination is very low; thus the benefit of immunization overweighs the negligible risk. The clinicians must be vigilant for diagnosis of hyperthyroidism, including Graves' disease in patients presenting with overactive thyroid signs or symptoms after recent COVID-19 vaccination.
